# Corrigendum

**DOI:** 10.1002/jcla.24900

**Published:** 2023-07-28

In Volume 37, Issue 3 (2023), the first affiliation was incorrect in the article titled “The role of 25(OH)D3 and circRNAs in early diagnosis of gestational diabetes mellitus” by Jinghui Zou, Yan Liu, Jun Shen, Aijiao Xue, Lulu Yan, and Yisheng Zhang. The first affiliated university Ningbo University Medical Center Lihuili Eastern Hospital name used by the hospital before, and now, the hospital has been renamed Ningbo Medical Center Lihuili Hospital.

The authors regret the preparation error and apologize for any confusion.

